# Harnessing Geospatial Artificial Intelligence (GeoAI) for Environmental Epidemiology: A Narrative Review

**DOI:** 10.1007/s40572-025-00497-4

**Published:** 2025-09-26

**Authors:** Hari S. Iyer, Seigi Karasaki, Li Yi, Yulin Hswen, Peter James, Trang VoPham

**Affiliations:** 1 Section of Cancer Epidemiology and Health Outcomes, Rutgers Cancer Institute, 120 Albany St. Tower 2, Office 8009, New Brunswick, NJ 08901 USA; 2 Epidemiology Program, Public Health Sciences Division, Fred Hutchinson Cancer Center, Seattle, WA USA; 3 Department of Epidemiology, University of Washington, Seattle, WA USA; 4 Department of Population Medicine, Harvard Medical School and Harvard Pilgrim Health Care Institute, Boston, MA USA; 5 Department of Biostatistics and Epidemiology, University of California San Francisco, San Francisco, CA USA; 6 Department of Public Health Sciences, Davis School of Medicine, University of California, Davis, CA USA; 7 Department of Environmental Health, Harvard TH Chan School of Public Health, Boston, MA USA

**Keywords:** Artificial intelligence, Geographic information systems, Environmental health, Epidemiologic methods, Big data, Data science

## Abstract

**Purpose of Review:**

Geospatial analysis is an essential tool for research on the role of environmental exposures and health, and critical for understanding impacts of environmental risk factors on diseases with long latency (e.g. cardiovascular disease, dementia, cancers) as well as upstream behaviors including sleep, physical activity, and cognition. There is emerging interest in leveraging machine learning and artificial intelligence (AI) for environmental epidemiology research. In this review, we provide an accessible overview of recent advances.

**Recent Findings:**

There have been two major recent shifts in geospatial data types and analytic methods. First, novel methods for statistical prediction, combining geospatial analysis with machine learning and artificial intelligence (GeoAI), allow for scalable geospatial exposure assessment within large population health databases (e.g. cohorts, administrative claims). Second, the widespread adoption of smartphones and wearables with global positioning systems and other sensors has allowed for passive data collection from people, and when combined with geographic information systems, enables exposure assessment at finer spatial scales and temporal resolution than ever before. Illustrative examples include refining models for predicting outdoor air pollution exposure, characterizing populations susceptible to water pollution, and use of deep learning to classify Street View image-derived measures of greenspace. While these tools and approaches may facilitate more rapid, higher quality objective exposure measures, they pose challenges with respect to participant privacy, representativeness of collected data, and curation of high quality validation sets for training of GeoAI algorithms.

**Summary:**

GeoAI approaches are beginning to be used for environmental exposure assessment and behavioral outcome ascertainment with higher spatial and temporal precision than before. Epidemiologists should continue to apply critical assessment of measurement accuracy and design validity when incorporating these new tools into their work.

**Supplementary Information:**

The online version contains supplementary material available at 10.1007/s40572-025-00497-4.

## Introduction

Geospatial data and geographic information systems (GIS) are essential tools for environmental health research, including the storage, processing, visualization, and statistical analysis of spatial data [1]. These core functions enable researchers to map areas with high burden of exposure and disease, incorporate spatial structure into models for prediction and causal inference, and inform efforts to identify environmental risk factors for disease [2–4]. Use of spatial data for environmental exposure assessment [5–7], disease mapping [6, 8–10], and planning and resource allocation [11, 12] has been a large and growing area of health research.

Two emerging trends have potential to markedly improve our understanding of how places and environmental contexts may influence human health and health behaviors. The first is the growing practice of combining geospatial analyses using large administrative population and environmental monitoring databases and research cohorts with artificial intelligence-based statistical predictions, or “Geospatial Artificial Intelligence” (GeoAI), which can process and analyze large amounts of geospatial data at scale, reducing researcher time and effort [13]. While some define GeoAI as methods that explicitly incorporate spatial dependence in machine learning algorithms [14], we broadened our review to cover applications of machine learning and artificial intelligence for exposure assessment and environmental epidemiology using geospatial data as predictors. More general overviews of GeoAI applications for health are available elsewhere [14–18]; here our focus is on GeoAI applications specifically for studying relationships between place-based environmental exposures and human health. Though GeoAI offers many opportunities to environmental epidemiologists (Box 1), it comes with its own set of challenges, such as understanding the assumptions made by these models and representative data for training and validation. The second trend is increasing volumes of “Big Data” for public health, which encompasses passively collected data from wearable devices smartphone-based GPS and behavioral tracking, and electronic health records [19]. These resources enable researchers to overcome earlier limitations of sample size and reduce costs of data collection. However, these new databases also pose their own challenges, including costs and availability of gold standard exposure data that can be used to train AI algorithms, outcomes, and relevant study features; and ethical implications arising from use of data that could identify an individual without their consent [20].

**Box 1** Examples of GeoAI with relevance to environmental epidemiology.

**Table:** 

**Classifying and predicting environmental features using satellite-derived remote sensing:** Satellite-derived Remote sensing data have used to train GeoAI methods for classifying land cover types or environmental features over differing spatial resolutions [21], identifying objects within an image [22, 23], cleaning remote sensing images (e.g. removing noise or errors introduced by atmospheric factors that interfere with sensors) [24], and identifying specific times or periods when the greatest change in environmental features is observed [25] **Urban planning-related contextual environment:** GeoAI approaches are being developed to integrate multiple streams of data generated by cities to improve lives of residents. For example, GeoAI is used by rideshare companies and public transportation authorities to more efficiently address demand by adjusting driver-passenger matching and traffic signals, respectively [26, 27], which could also reduce traffic-related pollution. GeoAI can predict areas at greatest risk of heat-related health issues by modeling the urban heat island effect [28]. Lastly, GeoAI is being used for sustainable energy consumption by adjusting power use to meet demand while reducing waste [29] **Earth systems analysis:** Building on earlier process-based Earth System Models, GeoAI have improved accuracy of short- and long-term climate predictions through integration of satellite-derived remote sensing data [30]. GeoAI has also been used to improve monitoring of greenhouse gas emissions through facility-level high-resolution mapping of carbon emissions [31]. In order to prevent harms from natural disasters, GeoAI is being incorporated into early warning systems for pollution and other extreme weather events [32, 33]	

Here, we describe the growing use of GeoAI for environmental epidemiologic exposure assessment and research. We then discuss how GeoAI applied to data from passive collection of health and behavioral data are capturing relevant exposures across an individual’s activity space, rather than just from their residence as was done in earlier eras. We discuss how geoAI approaches could contribute to recent advances in environmental epidemiologic methods and mixtures, along with ethical considerations required by investigators.

### Acquiring and Preparing Geospatial Data for GeoAI

GeoAI approaches require high-quality input data. Because specific geospatial data sources and data types have already been reviewed extensively in the context of public health research [13, 34–38], here we summarize considerations when acquiring and preparing input data sources for GeoAI applications in environmental epidemiology and exposure assessment focusing on the US, although most types of datasets described are available globally (Table 1).

**Table 1:** Major geospatial data used for GeoAI research with US examples

Data source	What is collected	Who collects	Types of environmental or contextual factors	Time period and frequency	Spatial scale	Example application of GeoAI with data source	Quality considerations	Example
Administrative	Disease registries, environmental monitoring, population demographics and economic factors	Government (local, national)	Socioeconomic factors (education, income, occupation), racial composition, poverty, air or water pollutants	1860-present Usually updated between 3–10 years	US: census block group, census tract, ZIP code tabulation area, county	Validate socioeconomic status measures estimated from Street View images of car make and model	Error in self-reported information, uncertainty, missing or erroneous percentages, harmonization of boundaries over time	National Census, American Community Survey, Demographic and Health Surveys
Satellite-derived Remote sensing	Satellite images, electromagnetic spectrum, aerosol optical depth	Government (national)	Land cover, greenspace, outdoor light at night, air pollution, aircraft imagery, building attributes, temperature	US: 1985-present Usually updated annually	Varies (30 m – 1 km are most common)	Feature detection and forecasting using self-attention models, enhance low-resolution satellite images, learning from unlabeled data	Accounting for surface reflectance, removing cloud cover, mosaics to stitch complete data	Landsat, MODIS, Copernicus
Street View Image	Panoramic images taken along road networks	Businesses and volunteers	Greenspace, neighborhood disorder	US: Varies (since 2007 for Google Street View)	Point locations	Classifying types of green space as perceived by people walking	May only be collected at a single time point,	Google Street View, Baidu, Mapillary (crowdsourced)

Administrative, satellite-derived remote sensing, and street view data are major sources of geospatial data available to environmental epidemiologists to characterize exposures. These sources are attractive for use in GeoAI because they offer extensive geographic coverage, raw data often are available at fine (< 1 km) spatial scales, and data are available over a long period of time (over twenty years in many cases) with annual or more frequent updates. Given the granularity of spatial and temporal data offered, these databases capture enormous variation in place-based characteristics that can be exploited for GeoAI methods to characterize social, built environment, and pollution exposures [16, 39–42]. 

Prior to applying GeoAI methods using these data sources, researchers should reflect on the completeness and accuracy of information contained therein. In addition, predictors may need to be normalized or transformed before applying GeoAI algorithms [43–46]. If predicting exposure by applying GeoAI algorithms in a novel dataset is the goal of the study, high quality labeled feature data are required. Certain databases may require further pre-processing. For example, satellite-derived remote sensing data often contain unusable or missing information due to cloud cover or other atmospheric anomalies that must be corrected using specialized algorithms [47, 48].

Researchers should also assess the appropriateness of a given geospatial database for their GeoAI study based on how data were sampled. Government, business, and volunteer-based sampling offer strengths and weaknesses in terms of coverage and precision. While government databases offer complete coverage of a population of interest, errors related to reporting and completeness may be more likely. Businesses may offer higher quality data, but may be smaller in scale and more costly to access. Volunteer-based data sources may offer more granularity than either businesses or government, particularly for select populations or geographies, but lack generalizability. Thus, epidemiologists should exercise judgment regarding the internal and external validity of input data sources and quality when determining an appropriate geospatial data source to use in a GeoAI algorithm [49].

### Narrative Review Methods

Our goal in this narrative review was to provide the reader with an overview of key concepts and emerging trends in the use of GeoAI in environmental epidemiologic and exposure assessment research, rather than providing a comprehensive review. We began by identifying papers in the past three years that applied deep learning and mobile phone-based big data approaches for assessing relationships between residential environment, pollutants, and lifestyles in clinical, cancer, and environmental health journals. We began with a focus on cancer because the use of geospatial analysis approaches has been a major research and funding priority by the US federal government in recent years [50].

In order to supplement our initial search, we obtained a list of peer-reviewed journal articles covering topics relevant to GeoAI and environmental health from PubMed using the “Advanced Search” tool with the search terms (geospatial artificial intelligence health) AND (environment), restricting to English language studies only. We then screened paper titles and abstracts to confirm that papers included in the review focused on environmental exposure assessment, human health, and application of statistical methods that incorporated spatial analysis and Machine learning or artificial intelligence-based methods to predict exposure, health outcomes, or relationships between the two. We excluded studies that were out of scope, reviews, abstracts, or did not focus on relevance to Human Health. Of 85 results, 43 were ultimately retained. This keyword search was complemented by snowball sampling of articles cited in references. These retained papers are summarized in Supplementary Table 1. Included papers focused predominantly on exposure assessment for air pollution and water quality (29/43, 66%), and compared different AI and machine learning approaches against one another using R^2^, Area Under the Curve (AUC), and Root Mean Squared Error (RMSE). The most common methods were eXtreme Gradient Boost (XGB) and Random Forest (RF), which generally produced the best predictions of exposure.

## GeoAI Combined with Big Data for Environmental Exposure Assessment

**Fig. 1:**
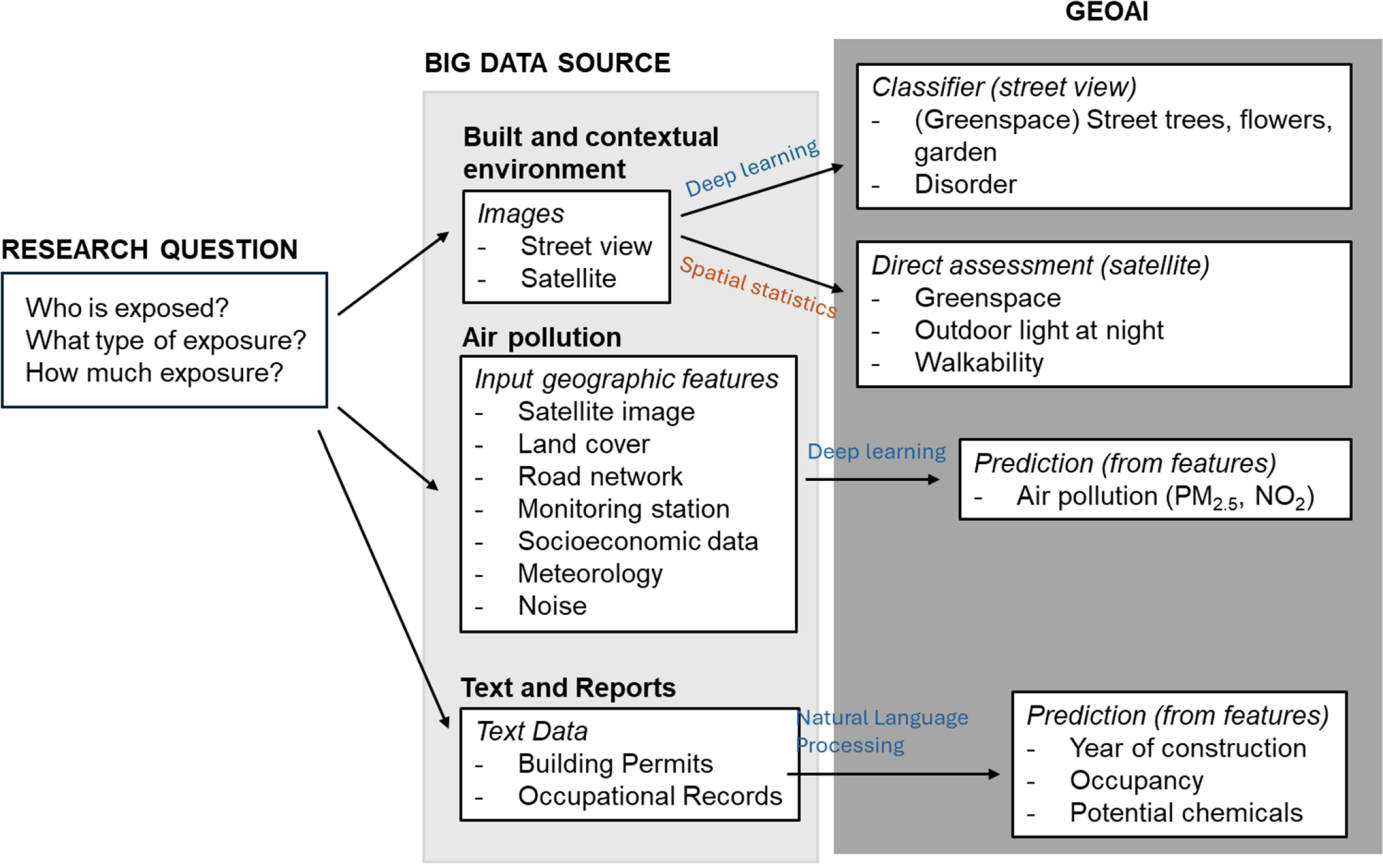
Offers a visual illustration of how GeoAI and Big Data sources are beginning to be used to advance environmental exposure assessment

For most environmental exposures, individual-level exposure measurement would be prohibitively expensive in large populations over long time periods. GeoAI is well-suited for exposure prediction, as it integrates spatial modeling and theory with large datasets and predictive algorithms to estimate where and for whom environmental exposures might pose the greatest risk or greatest benefit. When trained with high quality, representative data, deep learning algorithms can accelerate the laborious task of exposure assessment [51–53]. Geospatially derived exposures of air pollution, water pollution, and features of the built environment can be developed using geospatial AI approaches, and then used to map areas of high or low risk based on pollutant exposure and health [7] (Fig. 1).

### Enhancing Predictions of Ambient Air Pollution

Air pollution has been a major focus of GeoAI exposure modeling for environmental exposure assessments in epidemiologic studies [43, 48, 54–68]. Utilization of GeoAI methods has demonstrated improved predictive performance compared to earlier air pollution exposure models that relied on ground monitors only [69]. Exposure models have been developed for many air pollutants such as nitrogen dioxide [70, 71], ozone [57, 59], and ammonia [46] (Supplementary Table 1). Here, we focus on GeoAI methods for fine particulate matter (PM_2.5_), the most widely studied air pollutant [72].

Using GeoAI offers potential benefits over classical methods for air pollution prediction. First, these methods are able to account for complex characteristics of atmospheric mechanisms, including nonlinearity, interactions, and spatial and temporal autocorrelation [73], which can lead to better performance. Second, these models can be trained using publicly available satellite aerosol optical depth measures and low-cost local sensors and scaled across large populations and geographic areas, enabling studies of air pollution and health globally where government monitoring may be limited [43, 56]. While earlier statistical methods focused on outdoor air pollution only, studies are beginning to leverage available data to obtain estimates of exposure when traveling or when indoors [63]. The aforementioned GeoAI PM_2.5_ exposure models are associated with high predictive performance (cross-validation R^2^ ranging from 0.73–0.89) [73–76]. Further, these models have been used in epidemiologic studies that have contributed critical knowledge regarding the health effects of PM_2.5_ [77, 78]. Yet limitations of these methods include the requirement of large numbers of variables for prediction that may not be available for all study areas or time periods and high computing resource needs.

Di et al. (2016) developed a neural network-based hybrid model integrating satellite-derived remote sensing data on aerosol optical depth (AOD), chemical transport model outputs, land use, and meteorological variables [73]. Convolutional layers in the neural network were used for PM_2.5_ modeling to aggregate variable values from nearby grid cells or monitoring sites, which enabled the learning algorithm to determine the relative importance of variables while accounting for neighboring influences and potential interactions. Hu et al. (2017) created a random forest model incorporating geospatial data on predictors of PM_2.5_ concentrations not included in other exposure models such as percent impervious surface [74]. Compared to a neural network approach that involves a two-stage structure for training, a random forest approach implements a simpler one-stage structure. Di et al. (2019) established an ensemble PM_2.5_ exposure model integrating three machine learning algorithms (neural network, random forest, and gradient boosting) that incorporated over 100 variables using a generalized additive model and smoothing functions to account for nonlinear and/or geographically varying relationships [75]. Integrating PM_2.5_ predictions from multiple machine learning models allows for final predictions to take advantage of models that perform better in different settings based on location, concentration or time period, leading to overall improved model performance. Beyond the U.S., Shen et al. (2024) have optimized a deep learning residual convolutional neural network for global estimation of PM_2.5_ levels, which is able to identify and learn from images (e.g., spatial relationship of PM_2.5_ with predictors in its surrounding environment) [76].

Relatedly, accurate, reliable predictions of wildfire smoke (or extreme weather and climate events at large [79]) can support resource allocation, preparedness efforts, and early warning systems for vulnerable populations. Researchers have used methods ranging from gradient boosted trees [80] to ensemble-based deep learning [81] to generate high-resolution predictions of fine particulate matter in locations and time periods where direct measurements are unavailable. These approaches have also been used to model the spread of wildfire itself; Shadrin et al. (2024) used deep neural networks to predict wildfire spread [82].

### Augmenting Prediction of Drinking Water-Based Contaminants and Communities at Risk

GeoAI has also been used to estimate water quality, contaminants, and pesticides, enabling population-level estimates of exposed populations [45, 83–90]. Historically, the development of water pollutant models was labor intensive, requiring detailed maps of water pipes, well calibrated fate and transport physical models, and historical surveys of industrial contamination [91, 92]. Leveraging GeoAI methods can support linkages of water monitoring data to households that are most likely to receive water from a given distribution system, without requiring costly surveys and acquisition of private information about water systems. For example, the US EPA used a GeoAI approach to estimate community water system service areas across the US, leveraging public data on population characteristics and water system infrastructure to predict whether a given census block was more likely to be served by a water system or to procure its drinking water through other private means (e.g., domestic wells). Water system IDs were used to group together neighboring blocks predicted to be served by public water systems. For census block groups with multiple water system matches, they used random forest models to predict the most likely water system serving the majority of the census block group. Model validation using a test and train dataset found very high concordance (AUC = 0.9997 [93]).

GeoAI has also been used to identify areas where contamination is most likely to occur. Emerging contaminants, such as per- and polyfluoroalkyl substances (PFAS), pose significant challenges for drinking water management and provision and health [94–96]. With tens of thousand classes of PFAS, sampling and monitoring has proven to be both technically and financially difficult at scale. A recent study by Tokranov et al. (2024) used eXtreme Gradient Boosting (XGBoost), a tree-based machine learning approach, to predict the probability of PFAS detection in groundwater across the contiguous U.S. with an AUC = 0.83 [97]. The researchers trained their model using historical water quality data, the spatial distribution of known PFAS sources, and environmental and hydrogeological predictors, and validated their predictions using both *k*-fold cross validation and independent test datasets. A limitation of these studies is that sampling data are clustered in coastal areas and in Appalachia, with limited sampling in the central U.S.

Even long-term regulated contaminants like lead present significant monitoring challenges [98]. This is because lead contamination can be tested for and treated at the point of distribution, but contamination can occur between distribution and the point of exposurer [99]. Recent research has focused on predictive models to identify areas at high risk for lead contamination *past* the point of distribution. Studies by Huynh et al. (2024) applied GeoAI approaches for estimating lead exposure from drinking water in schools [100], with Huynh et al. applying microsimulation modeling approaches to predict potential high-risk levels in children through linkages with sociodemographic information from the US Census. Hajiseyedjavadi et al. (2020) investigated homes with high lead concentrations in tap water [101], applying spatial cross-validation in their machine learning algorithms to predict lead levels, which reduced potential bias in predictions arising from non-uniform spatial sampling of points.

### Improving Built Environment, Greenspace and Pollutant Exposures Using Satellite-Derived Remote Sensing

Satellite imagery captures a variety of information, including visible features of land (built environment, settlements, greenspace) and atmospheric elements (chemical composition of atmosphere, spectral bands of light). The breadth of built and contextual environmental factors captured can, when combined with GeoAI methods, allow for improved disease prediction and inference regarding drivers of morbidity in different geographic settings [62, 64, 102–108]. GeoAI enables use of social, built environment, land cover, meteorological, and environmental monitoring data to be used to assess correlations with different disease types.

An important application of these Big Data satellite instruments is to study potential impacts of exposures to nature (e.g., spending time in forests and parks) and health [109–111]. In the Nurses’ Health Study (NHS) and Nurses’ Health Study II (NHSII) prospective cohorts, Landsat-derived greenspace was appended to participants’ addresses in prospective studies of systemic inflammation [112], cognitive function [113], and depression [114], suggesting potential health benefits of residing in neighborhood environments with higher exposure to vegetation. Satellite-derived measures of greenspace from Landsat satellites were linked to residential ZIP codes among US Medicare Claims beneficiaries, revealing possible inverse associations with cardiovascular disease hospitalizations and possible increases in respiratory disease hospitalizations in urban areas [115]. Residential ZIP code-level greenness was associated with lower rates of Alzheimer’s Disease and related dementia hospitalizations [116]. 

Sentinel satellite data on air pollutants offers scientists in resource-limited settings with relatively weaker governmental regulation of environmental air pollutants to monitor changes in pollution associated with health outcomes. For example, studies in India [117] and Iran [118] demonstrate how academics are beginning to leverage Sentinel data to identify areas and communities who may be at heightened risks of high air pollution exposure, demonstrating that with sufficient technical capacity in use of GeoAI techniques, epidemiologists can produce estimates of health impacts of air pollution more efficiently.

### Detecting Street-Level Features of the Natural and Built Environment

While use of satellite-derived exposures has advanced the study of environmental exposures and health, they may not accurately reflect an individual’s interaction with their environment [119]. This limitation may be overcome through use of ubiquitous street-level images, which provide panoramic views of a point location that better captures an individual’s perception of their environment [107, 120–123]. Deep learning techniques, such as convolutional neural networks, can be applied to street-level images to identify environmental features such as greenspace, building density, and physical disorder that may impact health [124]. Outputs of these deep learning techniques can be used to detect built environment features, such as trees, grass, sidewalks, and urban disorder from eye-level views [124], which may be more strongly correlated with individuals’ own perceptions compared to residential satellite-derived measures [125]. Emerging analyses that apply street-view exposure metrics could further advance the field by elucidating, for example, the specific type of greenspace features that may drive health behaviors, mental health, and disease outcomes.

Street-level environmental exposure assessment through deep learning applied to street-level images have been used to study health effects of visible greenspace. For example, Yi et al. examined associations of different street-view greenspace components with adiposity measures (landscaping items such as flowers and plants) and cardiovascular health (trees), respectively, in Project Viva children—an eastern Massachusetts-based cohort [125, 126]. Yi et al. further examined over 350 million street-level images and found street-view trees were associated with lower incident depression over 17 years of follow-up [127]. Lastly, street-view trees were also found to be associated with a lower risk of Parkinson’s Disease among 45.6 million Medicare beneficiaries across the US [128]. Nguyen et al. (2019, 2021) [129, 130] applied computer vision techniques to over 16 million street-level images and found area with limited ground-level greenspace infrastructure exhibited higher rates of obesity and diabetes. These studies demonstrate how combining GeoAI with Big Data from Street View images can advance earlier work using satellite-derived greenspace measures, revealing specific forms of greenspace (e.g. street trees) that may offer the most benefits for health.

## Novel Big Data Sources for Acquiring Population Health Behavior Data

### Passive Data Collection to Describe, Map, and Predict Environmental Burden

Low-cost personal PM_2.5_ monitors [131], such as the Airbeam by Habitat Map (a community-based organization in Brooklyn, New York), are portable, able to track individual-level exposures over time, and enable easy data-sharing with researchers or community members [43]. Over 3 billion measurements have been taken using Airbeam. Users may contribute their monitoring data to public maps, which can supplement government monitoring databases, and be leveraged for communities to advocate for regulations [132, 133]. The Smoke Sense citizen science program of the EPA is another example of community-based monitoring and education regarding wildfires and ambient air pollution [134]. Users download an app and can test knowledge regarding protective behaviors, share data on pollution in their environment, and receive smoke and air quality alerts. These programs suggest that crowd-sourced and community-focused efforts to expand exposure monitoring can allow for population-based sampling, which may overcome limited generalizability of exposure distributions and effect estimates from occupation-based environmental epidemiology Big Data cohorts.

### Consumer Wearable Devices, and Smartphones for passive Health Behavior Data Collection

**Fig. 2:**
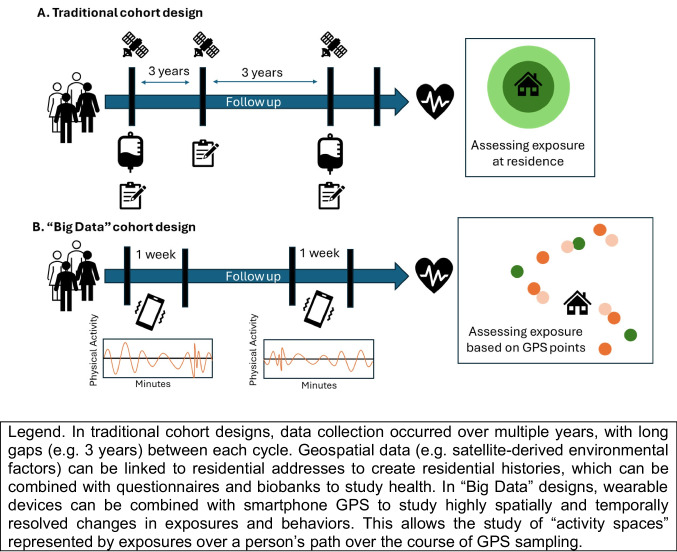
Traditional vs “Big Data” Cohort Design

Researchers are merging spatial datasets with accelerometry and consumer wearable devices, which allow high temporal resolution (e.g., 50 measures per second), objective data on movement, and other data streams, including light sensors and heart rate sensors [135, 136]. These devices can be used to derive validated measures of physical activity, sleep, and heart rate variability. Much of this work originated with research-grade accelerometry, including Actigraph devices [137], which researchers provided to participants for a sampling burst (e.g., one week) to derive health behavior metrics. More affordable consumer wearable options, including the Fitbit and Apple Watch, have enabled longer term follow-up of health behaviors [138]. Recently, more complex algorithms have developed over time to derive meaningful signals from wearable data, such as the type of physical activity or sleep stages These behavioral data can then be linked to environmental exposure data derived via location data collected from study participants (e.g. residential addresses or smartphone GPS data) to examine associations between their spatial exposures and objective health behaviors (Fig. 2).

Alongside research-grade accelerometry and consumer wearables, widespread use of smartphones has provided another opportunity for intensive data collection schemes, which an increasing number of studies have started using [139–141]. Compared to studies that rely on mailing wearables, smartphone-based collection of behavioral data is less burdensome and costly, allowing for longer follow-up periods due to secure uploading of near–real-time passive GPS and accelerometer data [142]. High accuracy smartphone GPS data can be merged with spatial datasets containing information on built and natural surroundings, noise levels, and air pollution to produce customized exposure metrics for environmental factors that change throughout the day over weeks and even months [143–146]. The smartphone accelerometer can take precise measurements through efficient sampling schemes that can provide an objective indicator of physical activity by identifying steps and cadence using advanced walk detection algorithms [147]. Smartphone apps can also be programmed to administer surveys on health behaviors or psychological health outcomes [148]. More importantly, these datasets can be linked at different temporal scales and sequences to examine the relationships between environmental and behavioral factors and chronic disease outcomes.

### Analyzing Social Media to Assess Responses to Environmental Hazards

The integration of online data sources, including social media and other digital platforms, can provide real-time insights into environmental and health-related exposures [65, 149]. Social media platforms offer rich geospatial data that can be leveraged for exposure assessment. X (formerly Twitter) enables researchers to analyze geotagged posts and monitor real-time environmental hazards. Hswen et al. (2019) demonstrated that geotagged tweets could be used to monitor air pollution exposure by correlating sentiment analysis (use of AI to attribute emotion and public opinion to social media discusions) with air quality index data [150]. Social media often provide real-time information on locations when an acute environmental disaster occurs, and allow government officials to share information with affected citizens, and for communities to share information with one another about the unfolding disaster and to let responders know where resources were needed [151]. Social media can also be studied following a disaster to understand how the public is reacting, and which individuals and institutions are most influential in sharing information [152]. Applying sentiment analysis using GeoAI approaches can reveal useful information for responders, such as when a study following Hurricane Matthew found that damage-related, rather than disaster-related tweets were more important predictors of need [153]. These examples reveal how social media Big Data sources can augment traditional environmental exposure studies by allowing for joint examination of spatial and social context, improving predictions of public understanding of where to find help and resources during a disaster [154].

Beyond X, Google search queries provide real-time assessments of health trends. Sadilek et al. (2020) demonstrated that Google search trends could predict Lyme Disease outbreaks, achieving a 92% correlation with CDC case data at the county level [155], revealing potential for GeoAI to support estimates of health and behavioral data for environmental epidemiologic research. Instagram and TikTok contribute to geospatial exposure assessment through location-tagged images and videos, allowing for evaluations of urban greenery, pollution sources, and built environments [156]. Facebook and Reddit facilitate community-driven discussions on environmental hazards, offering qualitative insights that complement quantitative monitoring systems [157].

Natural language processing and AI algorithms can be applied to social media and other text databases to serve as new sources of environmental exposure data [158]. A group of researchers in Malta were able to use natural language processing to extract features from 100,989 building permits into a geocoded database with building information to assess potential hazards, including year of construction, height and occupancy [159].

## Applications of GeoAI for Data Analysis and Interpretation

### Handling Spatially Correlated Data

Geospatial data often exhibit spatial autocorrelation whereby observations that are closer to each other are more similar than those that are further away [160]. This spatial dependence has implications for GeoAI algorithms when applied for exposure assessment and for causal inference in environmental epidemiologic studies [161].

When applying GeoAI algorithms for exposure assessment, spatial dependence of input predictors can be encoded into the algorithms themselves, referred to as “spatial embedding or spatial representation learning” [162]. This approach goes beyond the use of standard AI approaches discussed previously (e.g. greenspace in street view discussed in Section 2.4) by incorporating expert knowledge regarding spatial relationships between features that are being used to predict the exposure [163, 164]. For example, recurrent neural networks and transformer-based AI models to predict traffic patterns, an important contributor to air pollution, have been extended to incorporate spatial dependency using latitude and longitude to more precisely estimate changing traffic patterns [165, 166]. Spatial graph neural networks are alternative data models that do not rely on grid-based search, and can be augmented to incorporate expert knowledge regarding geographic and spatial relationships as nodes and spatial constraints in estimating weights when constructing the input graph [163].

Spatial dependence poses challenges to potential outcomes-based causal inference. Environmental epidemiologic studies relying on spatially-derived exposures are susceptible to unmeasured spatial confounding [167]. GeoAI could inform expert selection confounder sets, particularly when applying two-step methods involving prediction of environmental exposure conditional on covariates (e.g. inverse probability of treatment weighting (IPTW) or propensity scores) [168]. These machine learning and AI-based approaches for estimating propensity scores and IPTW could incorporate geospatial environmental and contextual factors, along with location-based parameters [163, 169, 170].

### Analyzing Complex Exposure Mixtures, Exposomics

The exposome is defined as the totality of environmental exposures across the life course [5, 171, 172]. Exposomics aims to comprehensively investigate the cumulative effects of physical, chemical, biological, and psychosocial influences that impact biological systems through leveraging data from interdisciplinary methodologies to enable discovery-based analysis of environmental determinants of health [173]. This aim is clearly lofty—GeoAI approaches can assist in reaching this goal by facilitating the analysis of high-dimensional, correlated datasets.

Mixture modeling provides methods to study the independent and aggregate effects of environmental exposures [174–176]. For example, Bayesian kernel machine regression (BKMR) models the health outcome as a smooth kernel function of the exposure variables adjusting for confounders, and accounts for collinearity using hierarchical variable selection [177]. There are also other mixture modeling methods including Bayesian Weighted Quantile Sum (WQS) regression and quantile g-computation [176, 178–181]. In addition, machine learning methods can be combined with mixture modeling to determine interactions between certain mixtures and/or mixture components. For example, Boosted regression trees can estimate H-statistics to rank the importance of exposure pairs [182, 183]. Signed iterative random forest in conjunction with WQS regression has been used to search for synergistic effects of chemical stressors on autism spectrum disorder [183, 184].Machine learning approaches based on penalization and shrinkage have been proposed to allow WQS to evaluate effects in opposing directions of environmental mixtures [185].

Challenges for exposomics studies, which can examine a large number of environmental exposures, include the lack of standardized approaches for selecting geospatial exposure models for a given environmental factor [175]. Use of AI-based approaches, which incorporate spatial predictors, can improve interpretability of findings and help screen large numbers of candidate exposome variables [186]. Mixture modeling methods are each associated with limitations that should be considered such as sensitivity of the posterior inclusion probabilities to tuning parameters in BKMR and loss of information due to data transformation to quantiles in WQS [187].

### Interpreting Model Parameters and Feature Importance

Most epidemiologic investigations seek to make inferences regarding what factors are most important or may cause the outcome of interest. A limitation of AI approaches is that the parameters used to predict exposures or outcomes are less interpretable than standard regression-based approaches [161]. In order to understand which predictors are most important, Shapley Additive exPlanations (SHAP) are commonly applied to examine the magnitude and influence of each geospatial and other predictors on the overall exposure prediction. In most papers we reviewed for air and water quality exposure assessment using AI methods, the authors provided SHAP beeswarm plots and reported the rankings of predictors [45, 46, 54, 57, 58, 67, 85, 149, 188].

## Challenges and Limitations when Applying GeoAI for Environmental Epidemiology

### Spatial and Temporal Issues

GeoAI tools may excel at predicting environmental phenomena relative to traditional methods, but cannot produce a conceptual model for an epidemiologic relationship with a given environmental exposure. Epidemiologists and exposure scientists must provide guidance regarding the physical and pathological processes that link exposure to outcomes. This requires scientists to continue considering the core principles of time, place, and population when designing epidemiologic studies.

Because environmental exposures and related spatial phenomena are so context-dependent, GeoAI algorithms are likely to perform poorly when applied in different geographic settings and different time periods as argued by Goodchild and Li [161]. Exposure models derived from AI models exhibit variable quality in predictions over larger geographic areas, which may lead to poorer performance in less populous areas and demographic groups [55–57, 84]. Some AI models such as recurrent neural networks struggle to model long-term trajectories because of the backpropagation process used in estimating weights [189] though alternative AI algorithms, such as Long Short-Term Memory can address some of these issues [190]. For a given research question, the input exposure data frequency and quality may determine the time period over which GeoAI algorithms can be used to predict exposure histories.

With respect to geographic context, statistical relationships between spatially defined exposures and outcomes are sensitive to the choice of spatial scale, referred to as the Modifiable Areal Unit Problem [191]. Investigators should specify clear conceptual models linking exposures and outcomes to determine the relevant spatial scale. Harmonizing exposure and outcome data collected at different spatial scales may involve areal interpolation that can introduce noise leading to loss of precision [192]. In addition, edge effects at boundaries of geographic areas can cause unstable predictions or introduce bias in epidemiologic studies [193]. In these settings, extending the boundaries using buffers can be used as a sensitivity test.

### Ethical Issues

Verifying input data quality for GeoAI is essential, because without human interpretation, decisions made by AI models may Yield unexpected or unwanted outcomes. A recent review of 91 Machine learning algorithms for clinical care, 87% reported bias for a socioeconomically disadvantaged group [194]. Application of an AI-based model within a hospital system exacerbated health disparities by preferentially excluding racialized minority patients and those from low-income neighborhoods from certain health services due to predicted increases in hospital stays [195]. These models may also have less accurate predictions in racialized minority groups [196]. In environmental epidemiologic studies, careful sampling strategies that ensure sufficient numbers of individuals across demographic and socioeconomic strata could enhance external validity of effect estimates and ensure that exposure measures are equally accurate across populations [197].

An assessment of the “bias and fairness” of the algorithm should be included when reporting results. These measures go beyond standard assessments of statistical accuracy such as calibration (how accurate predictions are with respect to observed exposures) and discrimination (how well the classifier algorithm separates exposed vs unexposed). Specifically, evaluations of fairness for AI algorithms reveal for whom algorithms provide more accurate vs less accurate predictions with respect to sociodemographic characteristics. Evaluating accuracy, positive and negative predictive values, and true and false positive rates across different groups can ensure that exposure assessment models are not inadvertently biased [55, 195, 198, 199].

Another ethical consideration of GeoAI-based exposure assessment relates to privacy and confidentiality. Federal regulations strongly restrict access to residential addresses for research because they may identify individual patients. However, displacement of geocodes by introducing random “noise” (geomasking), has been shown to reduce disclosure risks [200, 201]. Efforts have been made to avoid sharing sensitive data while allowing geocoding and spatial linkages to be done where PHI is stored and used for research. Examples include the Decentralized Geomarker Assessment for Multi-Site Studies (DeGAUSS) method by Brokamp et al. [202] AI models may eventually be able to create synthetic populations that preserve statistical relationships from the observed data, which could be analyzed without disclosing patient information [203].

### Measurement Error

Measurement error is a common source of bias in geospatial health studies, which occurs due to inaccuracies in geocoding [204], violations of assumption that residential addresses reflect personal exposure [205], and limitations in spatial resolution [141]. Even when methods like mobile GPS approaches are used for exposure assessment, the internal biologically active dose of exposure must be assumed rather than directly measured. GeoAI methods could be applied to evaluate measurement error in environmental epidemiology studies [55]. For example, methods involving machine learning have been developed to estimate the optimal (most strongly predictive) geographic buffer size for capturing the most relevant greenspace exposure on depression [206, 207], and spatial data could improve predictions. In addition, conformal prediction, a machine learning approach to quantify uncertainty of predictions, has been extended for spatial analysis allowing for more nuanced interpretation of AI-based predictions in spatial environmental contexts [208] by providing information about how precise or imprecise the GeoAI-derived exposure assessment may be, and therefore how much measurement error may be introduced when using the GeoAI-derived exposure.

## Conclusion

In summary, GeoAI offers exciting opportunities to improve exposure assessment and analysis for environmental epidemiology research. When combined with existing administrative, street view, or satellite data, GeoAI allows efficient exposure assessment and prediction for settings that have previously been difficult or costly to capture, such as perceived built environment and water contamination. GeoAI can also be integrated with passively collected data from wearable devices and high resolution GPS tracking to characterize exposures occurring beyond a participant’s residence, reducing measurement error. Lastly, GeoAI may improve performance of novel mixtures-based analysis approaches, and augment methods to correct for study biases. Alongside the promise offered by GeoAI, researchers should consider potential issues arising from poor quality training data, the timing and spatial coverage of training data and its relevance to the population of interest, and the need to maintain privacy and confidentiality when using participant address information.

## Key References

- Mai G, Xie Y, Jia X, Lao N, Rao J, Zhu Q, et al. Towards the next generation of Geospatial Artificial Intelligence. International Journal of Applied Earth Observation and Geoinformation. 2025 Feb 1;136:104,368.
  - ⚬ This review provides perspectives from the fields of remote sensing and geography of potential for GeoAI to improve classification and prediction of environmental features derived from satellite images.
- Di Q, Kloog I, Koutrakis P, Lyapustin A, Wang Y, Schwartz J. Assessing PM2.5 Exposures with High Spatiotemporal Resolution across the Continental United States. Environ Sci Technol. 2016 May 3;50(9):4712–21. 
  - ⚬ This paper provides a rigorous example of leveraging GeoAI and related machine learning approaches to improve prediction of fine particulate matter pollution.
- Montanari A, Fancello G, Sueur C, Kestens Y, van Lenthe FJ, Chaix B. A sensor-based study on the environmental determinants of sleep in older adults. Environmental Research. 2025 Jun 1;274:120,874.
  - ⚬ This paper leverages wearable devices and GPS to study associations of multiple environmental exposures with sleep, and found that neighborhood socioeconomic status was the strongest predictor of sleep quality.
- Yi L, Hart JE, Roscoe C, Mehta UV, Pescador Jimenez M, Lin PID, et al. Greenspace and depression incidence in the US-based nationwide Nurses’ Health Study II: A deep learning analysis of street-view imagery. Environment International. 2025 Apr 1;198:109,429.
  - ⚬ This study applied a deep learning model to classify street view images within a large prospective cohort study and found that visible trees were associated with lower depression.

## Supplementary Information

Below is the link to the electronic supplementary material.

Supplementary file1 (DOCX 29 KB)

## Data Availability

No datasets were generated or analysed during the current study.
