# Supplementary material for: Harnessing Geospatial Artificial Intelligence (GeoAI) for Environmental Epidemiology: A Narrative Review
Source: Curr Environ Health Rep. 2025 Sep 26;12(1):34. doi: 10.1007/s40572-025-00497-4 (PMC12474636; doi:10.1007/s40572-025-00497-4)
Supplement: Supplementary file 1 — Supplementary file1 (DOCX 29 KB) [file 40572_2025_497_MOESM1_ESM.docx]

**Supplementary Table 1. Summary of PubMed Search for Papers Containing GeoAI-Relevant Keywords**

| **Author** | **Year** | **Pollutant/Exposure** | **Type of study** | **Setting** | **AI/ML method** | **Geospatial data sources** | **Key Finding** |
| --- | --- | --- | --- | --- | --- | --- | --- |
| Ahn SH | 2023 | Groundwater microbes and chemicals | Exposure assessment | South Korea | Averaged neural network, RF, binning to create a grid | Safe Groundwater Project in Unsupplied Areas data from South Korean government, land use type, year of well, harmful inorganics and organics, microorganisms, substances affecting aesthetics, standards | Inadequate water standards ranging from 46% to 65% over study period, NN yielded the best classifications (97%-99%) |
| Babaan J | 2024 | NO_2_ | Exposure assessment | Taiwan | Ensemble Mixed Spatial Models and SHAP | Air pollution monitors, distance to airport/industrial source, land use, satellite greenspace, elevation, meteorology, population | Predicted NO_2_ with R^2^ >0.9 |
| Benà E | 2024 | Radon | Exposure assessment | Pusteria Valley, Italy | RF, SHAP | Soil Gas Radon Concentration from monitoring, thoron, carbon dioxide, terrestrial gamma dose rate, permeability, radon in groundwater, fault density, elevation | R^2^ for RF model was 0.93 for training, 0.47 for test |
| Chambliss SE | 2024 | PM_2.5_ | Measurement error | San Francisco, USA | Bayesian Additive Regression Trees | local mobile monitoring data obtained by Google Street View cars equipped with monitors, land use regression models, open street map local sources of pollutants, road density, population demographics from American Community Survey | Difference between local and national air pollution models was observed and could be explained by differences in local pollution sources and population demographic variables with BART. National models may underestimate pollution in non-White, low-income neighborhoods |
| Chen G | 2018 | PM_2.5_ | Exposure assessment | China | RFs, generalized additive model | Ground PM_2.5_ measurements from China National Environmental Monitoring Center, Satellite-derived aerosol optical depth, meteorological data, land cover data, satellite-derived greenspace and fire counts | RF had R^2^ ranging from 0.83-0.86 for daily, monthly, seasonal and lowest RMSE. However, model performance varied by geography |
| Chen J | 2024 | Pesticides | Exposure assessment | Global | RFs | Surface water nitrate model, BOD, surface water pesticide measures, climate, hydrology, human, soil, land cover, physiography | AUC for model for pesticide = 0.96. Populations with high potential risks of pesticides concentrated in Africa and Asia. |
| Chen TK | 2023 | Built environment | Epidemiology | Denmark | Segmentation-based convolutional NN | Landsat satellite image, Danish Civil Registration System, LiDAR | Higher proportion of building density associated with higher depression rates over 5 years |
| Chen X | 2025 | Water quality | Exposure assessment | Pearl River Basin, China | Ensemble model using decision tree, extremely randomized trees, gradient boosting, RF, XGB, stacking model, boosting-bagging model, SHAP | Monitoring data from rivers, water temperature, turbidity, chemical concentrations | R^2^ ranged from 0.92 to 0.58 across 6 different basins |
| Dahu BM | 2024 | Built environment | Epidemiology | Missouri, USA | Deep convolutional NN (image extraction), RFs for studying associations of obesity | Sentinel-2 satellite images to extract features using ResNet-50 | Predictions yielded adjusted R^2^ of 0.43 for predicting census tract obesity prevalence using linear regression, 0.47 using RF. |
| Durrani TS | 2024 | Fluoride | Exposure assessment | Quetta City, Pakistan | RF, SVM, classification and regression tree model | Groundwater monitoring data | CART had highest R^2^ of 0.73 in training, 0.51 in test |
| Hagedorn B | 2025 | Water quality | Exposure assessment | California, USA | NNs, K-nearest neighbor oversampling for regression, SHAP | US census, EPA Environmental Justice Screen, GeoTracker for water monitoring | R^2^ for random cross-validation was 0.97, R2 for optimized spatial cross-validation was 0.72 |
| Hossain M | 2024 | Water quality | Exposure assessment | West Bengal, India | Maximum likelihood machine learning, empirical bayes kriging | Groundwater monitoring, Landsat, geological maps | Declining groundwater quality in West Bengal |
| Hsu CW | 2025 | Ultrafine particulate matter | Exposure assessment | Miaoli, Taiwan | CatBoost Regressor, Gradient Boosting Regressor, LightGBM Regressor, RF Regressor, XGBoost Regressor, SHAP | Ultrafine particulate matter concentration from drones, land use and road network, 3D building, other pollutants (CO, NO, NO_2_, NO_x_, O_3_, PM_10_, PM_2.5_, SO_2_), meteorological variables, industrial sources, agriculture and food business, emission sources | XGBR had best performance (CV R^2^ = 0.83, RMSE was smallest) |
| Hsu CY | 2024 | Ozone | Exposure assessment | Taiwan | Ensemble mixed spatial model with SHAP | Air pollution monitoring data, meteorological data, land use, elevation, satellite-derived (MODIS) greenspace, buffers to capture distance to airports, industrial parks, garbage incinerators, power plants, population census, Chinee festivals, seasons | R^2^ for ozone was 0.91 for whole sample, with some regional variation and variation across time of ay |
| Li Z | 2022 | Ozone and NO_2_ | Disease prediction | Federal District and Fortaleza, Brazil | RF, long-short term memory, and LSTM with attention mechanism | Dengue case counts, total rainfall, mean temperature, mean relative humidity, mean satellite-derived greenspace | LSTM and LSTM-ATT outperformed RF models with substantially lower RMSE |
| Li Z | 2025 | Meteorological/climate | Exposure assessment | Southeastern China | RF | Hourly O_3_ and NO_2_ observations from local monitoring stations and NASA's Goddard Earth Observing System Composition Forecast, meteorological data, global atmospheric composition, land use data | CV R^2^ for ozone from day 1 to day 5 ranged from 0.63 to 0.43, R2 for NO2 from day 1 to day 5 ranged from 0.65 to 0.55 |
| Liang L | 2023 | PM_2.5_ | Exposure assessment | Texas, USA | Multi-scale, attention-enhanced convolutional NN | low-cost sensors for PM_2.5_, satellite-derived aerosol optical depth products and airborne LiDAR-derived 3D urban form, traffic from road length and distance to roads | Predicted PM_2.5_ with R^2^ > 0.9 |
| Lim CC | 2019 | PM_2.5_ | Exposure assessment | Seoul, Korea | Land use regression with RF and stacked ensemble | Air beam local monitors, road networks, land use, buildings, public amenities, transportation points, waste areas | Adjusted cross-validation R^2^ for RF (0.73) and SE (0.80) outperformed LUR (0.63) |
| Liu R | 2023 | PM_2.5_ and PM_10_ | Exposure assessment | United Kingdom | Light gradient boosting model | hourly PM_2.5_ and PM_10_ data, meteorologic factors, aerosol reanalysis, emission inventory, land cover data, road network, train data, anthropogenic activities, spatiotemporal weights | Good prediction from 2010 to 2019 0.71-0.85 R^2^ and fair R^2^ for daily measures from 1998 to 2009 (0.32-0.65) |
| Lotfata A | 2023 | Environment, contextual factors | Disease prediction | USA | Geographically weighted RF | BRFSS, American Community Survey, Landsat satellite greenspace, temperature and meteorological | Local R^2^ ranged from 0.22-0.95, average was 0.31 |
| Lu J | 2021 | PM_2.5_, built environment | Disease prediction | Beijing, China | autoregressive integrated moving average, multilayer perceptron, long short-term memory | PM_2.5_ monitoring data, meteorologic data, emergency room visit data from 10 comprehensive hospitals | ARIMA max R^2^ = 0.70, MLP max R^2^ = 0.80, LSTM of max v of 0.78. Overall LSTM had best accuracy |
| Lu QO | 2024 | PM_2.5_ | Exposure assessment | Tainan, Taiwan | Multilayer perceptron, SVM | Household sampling of PM_2.5_, questionnaires, outdoor air quality monitoring, land use inventory, road networks, night markets, number and density of industries, vehicle use and types, ratio of indoor vs outdoor PM_2.5_ and PM_10_ | Adjusted R^2^ for Hybrid LUR-SVM and Hybrid LUR-MLP (CV) were 0.79, and for PM10, same were 0.84 and 0.79. Ensemble Multilayer Perceptron was deemed best |
| Luo J | 2022 | Built environment | Exposure assessment | Zhengzhou, China | Image segmentation using fully convolutional NN | Baidu street view images, house price data | Spatial clustering of house price and green space using BSV |
| Otieno TA | 2025 | Meteorological/climate | Exposure assessment | Kenya | RF, XGB, SVMs | Remote sensing Landsat 7 and Landsat 8 for vegetation indices, precipitation data and meteorological data | RF was superior for Enhanced Vegetation Index R^2^ = 0.82), SVM performed best with Normalized Difference Vegetation Index (R^2^ = 0.69). XGB was not consistently superior to other methods |
| Pala D | 2020 | Built environment | Epidemiology | New York City | Deep NN model | Images from National Agriculture Imagery Program from 500 cities project, health data from CDC | Clusters were created based on greenspace, residential areas with small houses, industrial areas, residential with large buildings |
| Pavicic M | 2023 | Built environment | Disease prediction | USA veterans | Iterative RF | VA Million Veterans Program, climate variable (temperature, precipitation), 2019 American Community Survey, Alcohol and firearm businesses, | Alcohol and firearm density, social support variables, certain ancestry and marital status predict suicide attempts |
| Podgorski J | 2022 | Groundwater iron and manganese | Exposure assessment | Bangladesh | RF and generalized boosted regression modeling | Groundwater chemistry measurements, temperature, depth, climate, soil properties, land use at between 250m to 1km resolution | AUCs range between 0.76 to 0.80 |
| Podgorski J | 2020 | Water Arsenic | Exposure assessment | India | RF | Arsenic concentration measurements in India and border areas, climate, soil, land cover, lithology, topographic wetness, water table depth | AUC = 0.86, though most observations were taken from small set of countries, feature importance showed that soil variables were most important |
| Rachele JN | 2021 | Built environment | Epidemiology | Brisbane, Australia | Generative Adversarial Networks | HABITAT cohort, 200 census areas, 16,000 participants. Areal imagery, Google street view | Addition of vegetation was associated with high physical function, removal of buildings was associated with low physical function |
| Ren X | 2024 | Air pollution, built environment | Epidemiology | New Jersey, USA | Geostatistical models with flexible random components (Poisson, Negative binomial, Poisson mixed effect, Negative binomial mixed effect, Poisson Bersag-York-Mollie, Negative Binomial Bersag-York-Mollie, RF, XGB | COVID-19 case, socioeconomic status, air pollutants, proximity to industrial sites, transport-related noise, occupation and commuting, long-term care beds, restaurants and supermarkets | Poisson had highest R^2^, but lowest accuracy, NB and Poisson BWM spatial models had similar accuracy and performance to RF and XGB. |
| Sarigai | 2021 | Water quality | Exposure assessment | Guangzhou, China | LASSO | Sampling data of odorous polluted water, remote sensing ASD analytical spectroscopy, water properties | Factor analysis identified primary loadings of predictors. Adjusted R^2^ for factor 1 was 58%, adjusted R^2^ for factor 2 was 0.41, adjusted R^2^ for factor 3 was 0.40 |
| Shen H | 2019 | PM_2.5_, social mobility | Exposure assessment | Wuhan, China | Deep belief network learning model | PM_2.5_ monitoring data, social sensing data (traffic index, check-in (Tencent location Big Data), road network data, places of interest), remote sensing greenspace, meteorological data, elevation | Optimal 10-fold cross validation R^2^ was 0.83 |
| Talukder H | 2024 | Built environment | Disease prediction | Los Rios, Chile | XGB Model | Leptospirosis status in household survey, animals at household, rodents, Participant demographics, household animal characteristics, spatial variables including house density, building density, land cover, climate variables | Predicted seropositivity AUC of 92% |
| Tella A | 2022 | PM_10_ | Exposure assessment | Selangor, Malaysia | XGB, RF, K-nearest neighbor, Naïve Bayes | Air quality data from government, land use, satellite-derived greenspace, soil-adjusted vegetation index, land surface temperature, wind speed, elevation, slope, and road density | AUC was highest for RF and XGB models (0.99), but KNN (0.97) and Naïve Bayes (0.92) also performed well |
| Wang B | 2021 | PM_2.5_ | Exposure assessment | China | Geo-intelligent LSTM model for PM2.5 | Hourly PM_2.5_ concentration data within the China National Environmental Monitoring Center (CNEMC) website, satellite observation with TOA reflectance and areal optical density, meteorological conditions, satellite-derived NDVI (MODIS) | Geoi-LSTM with ordered inputs had R^2^ of 0.82 from cross-validation in station, yearly cross-validation R^2^ was 0.59. Superior R^2^ and RMSE compared to geographic weighted regression, RFs, linear mixed effects model, multiple linear regression |
| Wang S | 2024 | Social media | Epidemiology | China | Transformer, light gradient boosting machine, SHAP | Sina Weibo (social media), Baidu Streetview, Autonavi, shop reviews, real estate, COVID-19 case rates, mobility, population data | Models trained for perception had 88% accuracy. Social support was associated with higher happiness sentiment in social media |
| Wang Y | 2019 | Landslides | Exposure assessment | Zhejiang Province, China | SVMs, artificial NN, RF | Landslide inventory from field survey, Google Earth, elevation, meteorology, distance to roads, distance to rivers, distance to faults, remote sensing greenspace, land use, engineering geological type, meteorologic data | RF performed best with respect to accuracy, kappa index and AUC |
| Wong PY | 2024 | PM_2.5_ | Exposure assessment | Taiwan | Five machine learning models, stacking ensemble model, SHAP | PM_2.5_ monitoring data, NDVI from MODIS, distance to airports, thermal power plants, incinerators, elevation, census data/worship, season | Predicted PM_2.5_ R^2^ around 0.9 (slightly lower in dusk) |
| Wu CD | 2024 | Ammonia (NH_3_^)^ | Exposure assessment | Taichung, Taiwan | Generalized Additive Models and AutoML software that uses regression, tree-based, and gradient boosting machines that resulted in extra tree regression, SHAP | NH_3_ pollutant monitoring data, online measurement of other pollutants, wind direction, temperature, relative humidity, land use and land cover, emote sensing derived vegetation | R^2^ for GAM was 0.45 in test, whereas for ETR-AutoML was 0.60 |
| Xu J | 2023 | Built environment | Epidemiology | China | Convolutional NN | Baidu streetview image, hospitalizations for CVD | Used CNN to generate perception scores and correlated with CVD hospitalizations at block level. Certain perception scores were correlated with CVD rates in a sex-specific manner. |
| Xu Y | 2018 | PM_2.5_ | Exposure assessment | British Columbia, Canada | Bayesian Regularized NNs, SVMs, LASSO, Multivariate Adaptive Regressions splines, RF, XGB, Cubist | aerosol optical depth, land surface temperature, water vapor, satellite-derived greenspace (MODIS NDVI), albedo, High Planetary Boundary Layer, wind speed, elevation, distance to ocean | RF (R^2^ of 0.49), XGB (R^2^ of 0.46), and Cubist (R^2^ of 0.48) had superior performance |
| Yue X | 2022 | Built environment | Exposure assessment | USA | Convolutional NN | Google Street View, CDC PLACES, Census | Multiple built environment features from GSV predict counts of certain chronic diseases |
| Zhang A | 2022 | Built environment | Epidemiology | China | Fully convolutional NN with semantic segmentation | Baidu streetview image, hospitalizations for psychiatric disorders | Street enclosure index but not green view index is associated with mental health |
| Abbreviations: ANN=artificial neural network, BRFSS=Behavior Risk Factor Surveillance System, CDC=Centers for Disease Control, CVD=cardiovascular disease, LASSO=Least Absolute Shrinkage and Selection Operator, LSTM=long-short term memory, MODIS=Moderate Resolution Imaging Spctroradiometer, NDVI=Normalized Difference Vegetation Index, RF=Random Forest, SHAP=Shapley Additive exPlanations, SVM=Support Vector Machines, XGB=eXtreme Gradient Boost | | | | | | | |
